# Targeting Tumor-Associated Macrophages to Increase the Efficacy of Immune Checkpoint Inhibitors: A Glimpse into Novel Therapeutic Approaches for Metastatic Melanoma

**DOI:** 10.3390/cancers12113401

**Published:** 2020-11-17

**Authors:** Claudia Ceci, Maria Grazia Atzori, Pedro Miguel Lacal, Grazia Graziani

**Affiliations:** 1 Department of Systems Medicine, University of Rome Tor Vergata, Via Montpellier 1, 00133 Rome, Italy; claudia.ceci@uniroma2.it (C.C.); mariagraziaatzori2@gmail.com (M.G.A.); 2 IDI-IRCCS, Via dei Monti di Creta 104, 00167 Rome, Italy; p.lacal@idi.it

**Keywords:** macrophages, immune escape, metastasis, melanoma, PD-1, CTLA-4, PD-L1, immune checkpoint, VEGFR-1

## Abstract

**Simple Summary:**

Stimulation of the host immune responses, through the use of biotech drugs that remove a brake on the immune system (immune checkpoint inhibitors), is a current widely used strategy to treat a variety of advanced-stage tumors with impressive outcomes also in patients refractory to standard chemotherapy. However, as in the case of metastatic melanoma, many patients fail to achieve a long-lasting clinical benefit. The aim of this article is to provide an overview of the current scientific evidence concerning the role played by cells of the tumor micro-environment, and in particular tumor-associated M2 macrophages, on the innate or acquired resistance of melanoma to immune checkpoint inhibitors. A special focus will be given to potential therapeutic interventions capable of counteracting tumor ability to evade the control of the immune system in order to enhance the efficacy of immune checkpoint inhibitors.

**Abstract:**

Immune checkpoint inhibitors (ICIs) represent a promising therapeutic intervention for a variety of advanced/metastatic solid tumors, including melanoma, but in a large number of cases, patients fail to establish a sustained anti-tumor immunity and to achieve a long-lasting clinical benefit. Cells of the tumor micro-environment such as tumor-associated M2 macrophages (M2-TAMs) have been reported to limit the efficacy of immunotherapy, promoting tumor immune evasion and progression. Thus, strategies targeting M2-TAMs have been suggested to synergize with immune checkpoint blockade. This review recapitulates the molecular mechanisms by which M2-TAMs promote cancer immune evasion, with focus on the potential cross-talk between pharmacological interventions targeting M2-TAMs and ICIs for melanoma treatment.

## 1. Introduction

“Immune checkpoints” refer to a family of proteins expressed on the surface of T-cells, interacting with specific receptors/ligands located on antigen-presenting cells (APCs) or cancer cells, and inhibiting T-cell receptor (TCR)-mediated immune functions. Up-regulated during T-cell activation, the immune checkpoint molecules, such as programmed cell death 1 (PD-1), programmed cell death protein ligand 1 (PD-L1) and cytotoxic T-lymphocyte associated protein 4 (CTLA-4), prevent an excessive immune response, potentially leading to tissue damage or to the establishment of an autoimmune disease. Immune checkpoint inhibitors (ICIs) allow the adaptive immune system to overcome this “turn-off” signal and to maintain an effective immune surveillance against cancer cells.

In the last decade, different monoclonal antibodies (mAbs) targeting immune checkpoints have been developed, i.e., pembrolizumab, nivolumab and cemiplimab, directed against PD-1; atezolizumab, durvalumab and avelumab, which target PD-L1; ipilimumab and tremelimumab, specifically recognizing CTLA-4. Indications of ICIs currently approved by the Food and Drug Administration (FDA) and European Medicines Agency (EMA) are reported in Table 1.

Unfortunately, data accumulated in recent years suggest that the clinical efficacy of ICIs is confined to a limited percentage of cancer patients. Furthermore, certain tumor types, including pancreatic, colorectal, ovarian cancer, show little benefits or are completely refractory to therapies based on immune checkpoint blockade [1]. Therefore, ICIs are not always able of efficiently reactivate exhausted tumor-specific T-cells and to restore a proper cancer immune surveillance [2], due to intrinsic or acquired mechanisms of resistance still not fully understood.

Little information is presently available concerning the potential interactions between ICIs and components of the tumor micro-environment (TME). Among the cell populations extensively recruited in the tumor mass, tumor-associated macrophages (TAMs) are known to hamper cancer patient’s response to traditional chemotherapy, and a growing literature shows their involvement in the failure of the anti-tumor immune surveillance, as well as of immunotherapy with ICIs.

Aim of this review is to recapitulate the pro-tumor functions of TAMs, in particular the molecular mechanisms by which TAMs polarized toward the M2 phenotype promote cancer progression and immune escape. A special focus is provided on the preclinical evidence suggesting TAMs involvement in melanoma immune evasion, and on promising clinical investigations combining TAMs targeting molecules with ICIs for metastatic melanoma treatment.

## 2. From Circulating Monocytes to Tumor-Associated Macrophages: Characterization of a Tumor-Sustaining Population

**Figure 1:**
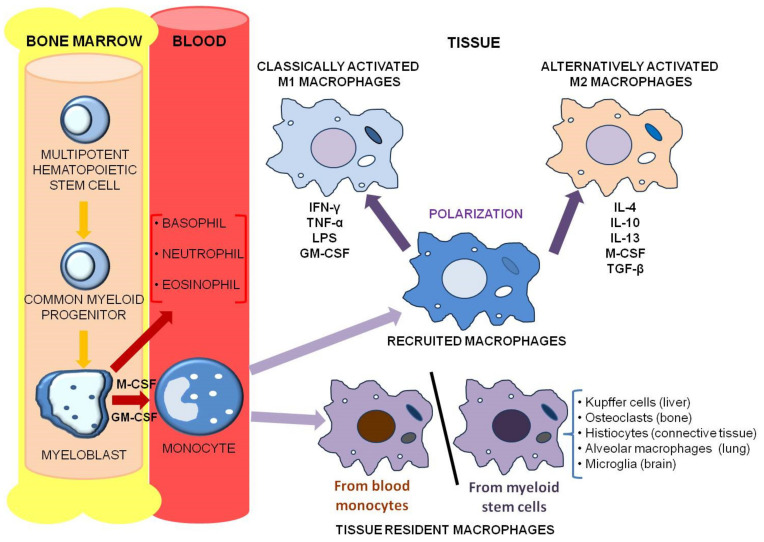
Ontogeny of human tissue macrophages. Macrophages are a subtype of white blood cells, originating from bone marrow progenitors. Multipotent hematopoietic stem cells generate common myeloid progenitors, which in turn give rise to myeloblasts. Monocytes, derived from myeloblasts (which are also the precursors of basophils, neutrophils and eosinophils), are released from the bone marrow into the blood circulation, and, within a few days, they accumulate in various tissues throughout the body, representing a storage reservoir for the production of tissue macrophages. Resident tissue macrophages are also formed during the embryonic development, independently of blood monocytes. Tissue macrophages orchestrate their immune function by a polarization process towards two different phenotypes: M1, i.e., classically activated or inflammatory phenotype, and M2, i.e., alternatively activated or anti-inflammatory phenotype. See text for further details.

First discovered late in the 19th century by Ilya Metchnikoff, macrophages are a subtype of white blood cells belonging to the mononuclear phagocyte immune system, which includes bone marrow progenitors, blood monocytes, and tissue macrophages. Monocytes behave as macrophage precursors: they are released from the bone marrow into the blood circulation and then accumulate in various tissues (Figure 1), forming a storage reservoir for the production of mature immune cells. Moreover, resident tissue macrophages are formed during the embryonic development and persist in adulthood, independently of blood monocytes [3,4].

Based on their localization and function, macrophages are grouped into different subpopulations, including specialized tissue-resident macrophages such as osteoclasts (bone), alveolar macrophages (lung), histiocytes (interstitial connective tissue) and Kupffer cells (liver). Secondary lymphoid organs also possess their own macrophages, which exert specific functions, including marginal zone macrophages in the spleen and subcapsular sinus macrophages in lymph nodes. Specialized macrophage subpopulations are also present in the brain (microglia), eye and testes, where they play central functions in tissue re-modeling and homeostasis [3].

Tissue-specific macrophages mediate non-specific defense or innate immunity, through the ingestion of foreign material (being able to phagocytize viruses, bacteria, and other foreign particles) and the recruitment of additional macrophages from their circulating precursors, processes aimed at eradicating an infection or ensuring recovery from an injury [3]. Furthermore, macrophages act as APCs: they display degraded foreign antigens on their cell surface, in association with major histocompatibility complex class II (MHC-II) molecules. This process activates T-cells, thus initiating the specific defense, or adaptive immunity mechanisms. Finally, macrophages exert an immune modulatory role by secreting various signaling molecules or cytokines/chemokines, which control other immune cell functions [5].

A key feature of macrophages, through which they can exhibit the required phenotype to orchestrate a functional response to specific stimuli present in their micro-environment, is plasticity: they can switch from one phenotype to another thanks to the polarization process. Based on gene expression, surface molecules and biological metabolites produced, macrophages can be polarized towards two distinct phenotypes: M1, i.e., classically activated or inflammatory subtype, and M2, i.e., alternatively activated or anti-inflammatory subtype.

The M1 macrophages are typically activated by Th1 cytokines, such as interferon-γ (IFN-γ) and tumor necrosis factor-α (TNF-α), or by recognition of bacterial lipopolysaccharide (LPS); in turn, they secrete high amounts of pro-inflammatory cytokines, such as TNF-α, interleukin-1 (IL-1), IL-6, IL-12, and IL-23. Functionally, M1 macrophages contribute to the removal of infectious pathogens, through activation of the nicotinamide adenine dinucleotide phosphate (NADPH) oxidase system, and consequent production of reactive oxygen species (ROS). Overall, M1 macrophages exert anti-microbial and anti-tumor activity, mediate ROS-induced tissue damage, and hamper tissue regeneration and wound healing.

Conversely, M2 polarization is induced by Th2 cytokines (e.g., IL-4, IL-13, IL-10) as well as by glucocorticoids. In turn, M2 macrophages have an anti-inflammatory cytokine profile, which is characterized by high production of IL-10 and transforming growth factor-β (TGF-β). Functionally, M2 macrophages have a potent phagocytic capacity that allows them to scavenge the debris of apoptotic cells, promote tissue repair and wound healing, and possess pro-angiogenic and pro-fibrotic properties. Therefore, M2 cells take part in the dampening of inflammation and coordinate tissue repair and re-modeling [5,6].

In addition to vitally important roles in the maintenance of tissue homeostasis, anti-pathogenic defense and inflammatory responses, macrophages also play a pivotal role in the onset and evolution of various diseases, such as autoimmune disorders, atherosclerosis, and cancer [7,8].

The malignant evolution of cancer is sustained by a complex cellular system, including leukocytes, fibroblasts, and vascular endothelial cells, all components of the TME. In particular, tumor cells cooperate with immune cells in mounting the inflammatory response representing a typical hallmark of tumorigenesis, and TAMs represent the major infiltrating immune cell population within the TME. Usually, TAMs derive from hematopoietic bone marrow precursors or circulating monocytes, recruited in the tumor tissues: chemokines, cytokines, growth factors and other proteins, secreted by tumor and immune cells, are able to assist the monocytes transmigration through endothelial cells of blood vessels, and their entering into the tumor tissues, where they are then induced to differentiate into TAMs. In some cases, a local, tumor-related proliferation of this macrophage sub-population has been also described [9,10]. Signals produced by tumor cells are crucial not only in recruiting TAMs, but also in inducing the M2-polarization and controlling their permanence in the TME. Chemokines such as CCL-2 (C-C motif chemokine ligand-2, also known as monocyte chemoattractant protein-1, or MCP-1), CCL-5, CXCL-12 (C-X-C motif chemokine ligand-12), the growth and differentiation factor M-CSF (macrophage colony-stimulating factor or colony-stimulating factor 1, CSF-1), cytokines and the vascular endothelial growth factors (VEGFs), are all examples of signaling molecules produced by tumor cells that act as recruiting and differentiating factors for M2-TAMs [11,12,13]. Of note, TAMs directly participate in CCL-2 and IL-10 production, suggesting the existence of an autocrine amplification loop involved in their tumor-related recruitment and polarization [14]. In in vitro three-dimensional models, IL-10 has been shown to be released also by melanoma cells leading to the induction of an M2-like phenotype in myeloid cells [15]. Furthermore, in melanoma cells, overexpression of bcl-2 correlated with the tumor ability to reprogram macrophage polarization toward M2 through bcl-2-dependent IL-1β production [16]. Modifications of the TME further increase the heterogeneity of M2-TAMs; even in the same tumor model, different subtypes of M2-TAMs could be distributed in different regions, i.e., M2a-, M2b-, and M2c-TAMs. IL-4 and IL-13 are the Th2 cytokines that typically induce M2a macrophages; LPS and IL-1 promote the differentiation of M2b-TAMs; IL-10 and glucocorticoids can elicit the M2c form of active macrophages. In turn, the different subtypes of M2-TAMs, induced by different tumor-derived factors, correspond to various grades of immunosuppressive functions: M2a-TAMs inhibit the Th1 but activate the Th2 immune response, while both M2b and M2c macrophages exhibit general anti-immunity effects [17].

Once recruited and polarized by cancer cells, M2-TAMs have an important influence on different aspects of tumor initiation and progression; consistently, TAMs infiltration often correlates with an unfavorable prognosis [9,10,14]. In particular, TAMs contribute to tumor initiation by secreting pro-tumorigenic signaling molecules, including TGF-β, TNF-α, ILs, M-CSF and CXCLs [14]. Furthermore, TAMs are the major source of growth factors in the TME, such as VEGF-A, placenta growth factor (PlGF), epidermal growth factor (EGF), fibroblast growth factor (FGF), which all support cancer cells proliferation and survival [18]. TAMs-derived cytokines, such as IL-23 and IL-17, have been shown to sustain tumor-elicited inflammation, which in turn drives tumor growth [18]. Moreover, macrophages are involved in the extracellular matrix (ECM) re-modeling and induction of angiogenesis, thanks to the production of matrix metalloproteinases (MMPs) and other factors. In fact, TAMs-derived VEGF-A, PlGF, CXCL-8 and prokineticin (Bv8) are all crucial proteins involved in cancer cell invasiveness and neo-angiogenesis, both processes required for tumor cell migration to distant sites through blood circulation, and metastases formation [18,19,20,21]. A recent study suggested a link between TAMs and the invasive potential of human gastric cancer cells, through the involvement of the pro-inflammatory enzyme cyclooxygenase-2 (COX-2) and MMP9 expression. The co-culture of in vitro polarized M2 macrophages with cancer cells, in a trans-well system, resulted in an increased COX-2 expression and cancer cell invasion, abrogated through the pretreatment with a COX-2 siRNA or with the COX-2 inhibitor celecoxib. Moreover, COX-2 inhibition was shown to block the promoting effect of macrophages on MMP9 expression [22].

Finally, crucial in tumorigenesis promotion by M2-TAMs is their role as immunosuppressive mediators, being able to inhibit the anti-tumor responses orchestrated by the Th1-mediated adaptive immunity [23,24] and to sustain the less destructive Th2 type immune responses, which may support the development of cancer cell resistance to ICIs. In fact, the success of an ICIs-based treatment requires CD8+ T-cells to be fully cytotoxic against tumor cells, a condition hampered by the immunosuppressive potential of M2-TAMs, being able to down-regulate T-cell activation and proliferation [25,26] through different mechanisms. Clinical evidence exists demonstrating that the tumor immune contexture, i.e., the density and phenotype of tumor-infiltrating immune cells, critically contributes to the efficacy of ICIs therapy. By using melanoma patients’ tissue samples collected before and during anti-PD-1 treatment (pembrolizumab), it was demonstrated that a higher frequency of pre-existing intratumoral CD8+ T-cells correlated with clinical responsiveness to anti-PD-1 therapy, in terms of radiographic reduction of the tumor size [27]. Another study, investigating renal cell carcinoma tissues from patients treated with a combination of anti-PD-L1 and anti-VEGF-A mAbs (atezolizumab and bevacizumab, respectively) revealed a correlation between a high T effector gene signature and an improved overall response rate and progression-free survival, while a high myeloid inflammation gene signature was associated with a reduced progression-free survival [28]. Furthermore, in advanced solid tumors the M1/M2 macrophage ratio score together with the tumor mutational burden and CD8+ scores were all predictors of response to ICIs [29].

## 3. Tumor-Associated Macrophages and Anti-tumor Immune Surveillance Evasion

**Figure 2:**
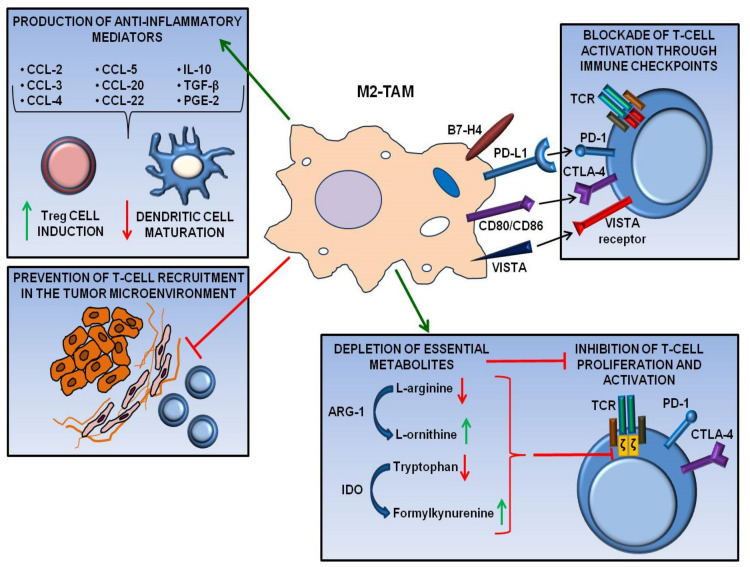
Mechanisms involved in the suppression of anti-tumor immunity mediated by TAMs. Immunosuppressive mechanisms supported by TAMs include: production of anti-inflammatory cytokines and chemokines and other inflammatory mediators that sustain Treg differentiation and hamper dendritic cell function; blockade of T-cell activation through the interaction with inhibitory immune checkpoints; depletion of essential metabolites for T-cell proliferation, such as arginine and tryptophan, due to the expression of specific metabolic enzymes (arginase-1, ARG-1, and indoleamine 2,3-dioxygenase, IDO, respectively); physical hindrance of T-cell recruitment in the TME. See text for further details.

Several mechanisms have been identified through which TAMs suppress anti-tumor immunity and may hamper ICIs activity, thus promoting cancer progression and resistance to immunotherapy [30]. In particular, it has been suggested that M2-TAMs inhibit cytotoxic T-cell function by producing anti-inflammatory cytokines, depleting essential metabolites for T-cell proliferation, and turning off T-cell activation through interaction with inhibitory immune checkpoints (Figure 2).

IL-10, prostaglandin E2 (PGE2), and TGF-β are examples of signaling molecules, produced by M2-TAMs under the influence of tumor-derived factors, which inhibit T-cell-mediated immune responses and contribute to the establishment of a self-propagating immunosuppressive TME [30].

IL-10 plays a crucial role in dampening anti-tumor immunity by suppressing the activity of different immune cells, eventually leading to the inactivation of effector T-cells [31]. In detail, TAMs-derived IL-10 inhibits APCs function [32], suppresses intratumoral dendritic cells (DCs) maturation, and reduces IL-12 production by DCs, thereby limiting cytotoxic T-cell activity [33]. Furthermore, IL-10 can directly down-regulate the activation of CD8+ T-cells, by increasing the expression of a glycosyltransferase that promotes N-glycan branching of surface glycoproteins. This event physically prevents CD8 protein and TCR co-localization and reduces the antigen sensitivity of CD8+ T-cells [34].

PGE2, a COX-2 product acting as a molecular mediator of inflammation and known to be involved in macrophage M2 polarization [35], contributes to suppress the cytotoxicity of natural killer (NK) cells and CD8+ cytotoxic T lymphocytes (CTLs). Moreover, PGE2 induces the expression of Foxp3, a transcription factor that stimulates the differentiation of immunosuppressive regulatory T-cells (Tregs) from naïve T-cells [36]. Another important immunosuppressive effect of PGE2 is the inhibition of the production by monocytes and DCs of CCL-19, a key chemokine that recruits naïve T-cells and activates effector T-cells [37]. Finally, through inhibition of IL-2 signaling, PGE2 promotes a switch from Th1 to Th2 immune responses [38], the first favoring cellular immunity by stimulating IFN-γ and TNF-α production, and, consequently, the cytotoxic activities of macrophages and CTLs.

M2-TAMs-derived TGF-β contributes to immune evasion by affecting both the adaptive and the innate immune responses, as assessed in many tumor types [39,40]. In metastatic urothelial cancer, TGF-β expression was associated with the exclusion of CD8+ T-cells from the tumor parenchyma, and with their delocalization in the fibroblast- and collagen-rich peritumoral stroma [41]. In colorectal cancer, increased TGF-β levels in the TME not only promoted T-cell exclusion but also blocked the acquisition of the Th1 effector phenotype [42].

Among chemokines, macrophages produce CCL-2, CCL-3, CCL-4, CCL-5, CCL-20, and CCL-22 that recruit Tregs to the TME and sustain their survival [43], with consequent inhibition of effector T-cell function.

By secreting arginase 1 (ARG-1) in the TME, M2-TAMs are also able to deplete arginine reservoir, a metabolite with a crucial role in T-cell proliferation and activation [44,45]. Lactic acid produced by tumor cells, known to exert a critical role in inducing M2-like polarization of TAMs, is a key player in promoting ARG-1 expression by macrophages [46]. ARG-1 metabolizes L-arginine to L-ornithine and other anti-inflammatory products, such as urea. L-ornithine, in addition to promote tissue re-modeling and wound healing [47], stimulates cancer cell proliferation, while L-arginine depletion reduces the expression of CD3 ζ-chain in the TCR complex, impairing effector T-cell-mediated responses to tumor antigens [48,49]. Furthermore, by up-regulating ARG-1, M2-TAMs also deplete the arginine pool for inducible nitric oxide synthase (iNOS), another enzyme that uses arginine to produce nitric oxide (NO), an important mediator of the immune responses against parasites and cancer [50].

Modulation of tryptophan metabolism is another way to affect the immune functions: both human and murine M2-TAMs overexpress indolamine 2,3 dioxygenase (IDO), an enzyme which converts tryptophan to formylkynurenine, and significantly decreases tryptophan availability for T-cells [51,52]. Furthermore, tryptophan depletion induces the stress kinase general control nonderepressible 2 (GCN2), which in turn down-regulates the expression of the CD3 ζ-chain in the TCR complex of CD8+ cytotoxic T-cells, and inhibits the differentiation of Th17 cells (IL-17 producing T-cells, generally considered to be positive regulators of the immune responses) [53,54]. In addition, kynurenine itself is a potent suppressor of T-cell function, since it can induce T-cell death or interfere with TCR signaling.

TAMs-induced immune suppression can be also mediated by the expression of PD-L1/PD-L2 and CD80/CD86, the ligands of the immune checkpoint inhibitory receptors PD-1 and CTLA-4, respectively [2,43,55,56]. Moreover, TAMs can sequester anti-immune checkpoint mAbs through the Fcγ receptor present on their cell surface, preventing the interaction of the antibody Fab regions with the target [57]. Indeed, in vivo imaging studies in different murine cancer models demonstrated that after intraperitoneal administration, an anti-PD-1 mAb co-localized with tumor-infiltrating T-cells at early time points, being then captured by TAMs [57]. Other immune checkpoint ligands expressed by TAMs, with a potential direct suppressive effect on tumor-infiltrating T-cells, are B7-H4 (also known as B7x, B7S1 or VTCN1) and V-domain Ig-containing suppressor of T-cell activation (VISTA, also known as PD-1H, B7-H5, DD1α) [58,59,60]. Cells expressing B7-H4 may negatively modulate the immune response by inhibiting T-cell proliferation and production of cytokines [61]. Remarkably, B7-H4 expression on TAMs correlated with the clinical stage in cancer patients [62]. VISTA, instead, is an immunosuppressive molecule expressed either on cells of the myeloid and lymphoid lineages (it seems to acts both as a ligand on APCs and as an inhibitory receptor on T-cells) that reduces T-cell proliferation and cytokine production, while sustaining Tregs function [63]. Consistently, VISTA has been proposed as an independent negative prognostic factor for multiple cancers, among which primary cutaneous melanoma. In fact, a recent study demonstrated a strong correlation between VISTA expression and tumor infiltration by myeloid cells and PD-1+ inflammatory cells. Interestingly, VISTA levels negatively correlated with patients’ survival [64]. Unlike the other better characterized immune checkpoints (PD-1, CTLA-4), induced at different stages after immune cells activation, VISTA is constitutively expressed. This property suggests an important homeostatic role of VISTA in regulating the immune system and qualifies VISTA as a promising target of cancer immunotherapy [65]. Modulation of both innate and adaptive immunity, obtained through an antibody targeting VISTA, slowed tumor growth in murine cancer models [66] by promoting a pro-inflammatory TME that favored T-cell infiltration. Furthermore, a recent study showed that VISTA-deficient myeloid cells presented a reduced chemotactic ability and that tumors grown in VISTA-deficient mice were markedly devoid of macrophages [67].

Still unknown is the mechanism through which M2-TAMs hamper anti-tumor immunity by physically preventing CD8+ T-cells from being properly recruited in the TME [68,69]. Fibrosis could represent a possible condition allowing TAMs to inhibit T-cell accumulation within the tumor mass: through interaction with fibroblasts, macrophages are known to actively participate in tissue re-modeling, inducing collagen synthesis and secretion [70]; furthermore, by producing granulin, M2-TAMs were shown to remodel the ECM [71] and induce fibrosis in the tumor stroma [72,73].

## 4. Melanoma and Immune Checkpoint Inhibitors

Melanoma is a potentially fatal skin malignancy, with a continuously increasing incidence worldwide: currently, lifetime risk of developing melanoma is 1 in 63 in the US and other western countries, and melanoma-related deaths represent 73% of skin cancer-related deaths. When diagnosis is performed at an early/localized stage, melanoma is manageable by surgery, with a 5-years relative survival rate of 98%. Conversely, even if several treatment options are now available, the prognosis of melanoma diagnosed at an advanced or metastatic stage is still poor [74].

A typical feature of malignant melanoma is a significant immunogenicity, mainly due to a high UV-driven mutational burden [75] that causes overexpression of tumor-specific antigens and promotes a favorable antigen specific immune response [76,77]. Nevertheless, melanoma can evade the anti-tumor immune control, thus becoming a highly aggressive metastatic malignancy [78].

ICI-based therapies, targeting CTLA-4, PD-1 and PD-L1, have markedly improved the clinical outcome of patients with advanced/metastatic melanoma [79]. The most recent clinical guidelines on melanoma management consider the anti-PD-1 agents nivolumab and pembrolizumab, alone or in combination (nivolumab) with the anti-CTLA-4 agent ipilimumab, as first-line options to treat unresectable stage III (locally advanced) and IV (with distant metastases) melanoma [80]. The same anti-PD-1 agents are also prescribed in an adjuvant setting for completely resected melanoma with lymph node involvement or metastatic disease [81] and are currently under investigation as neoadjuvant treatment [82,83,84] for resectable stage III melanoma. Finally, the anti-PD-L1 atezolizumab was recently approved by FDA for BRAF V600 mutated, advanced melanoma in combination with the BRAF and the mitogen-activated protein kinase (MEK) inhibitors vemurafenib and cobimetinib, respectively (see also Table 1).

Currently, 5-years overall survival rates of 44% [85] and 41% [86] were reported with nivolumab and pembrolizumab, respectively. The combined immunotherapy of unresectable or metastatic melanoma with nivolumab plus ipilimumab [87] resulted in a 5-years survival rate of 52% (vs 44% for nivolumab and 26% for ipilimumab) and an objective response rate of 58% (vs 45% and 19% for nivolumab and ipilimumab alone, respectively) [85]. However, a significant toxicity is experienced: grade 3 and 4 adverse events have been reported in up to 59% of patients treated with the mAb combination, compared to 23% and 28% of patients treated with nivolumab or ipilimumab as single agents, respectively [88].

Pembrolizumab and low-dose ipilimumab combination, although not yet approved, achieved a comparably high objective response rate (in 61% of patients) and an estimated 1-year overall survival of 89% [89]. Furthermore, a lower toxicity was assessed, compared to nivolumab plus ipilimumab combination: grade 3 or 4 drug-related adverse events occurred in 27% of patients. First data from an ongoing phase 2 clinical trial confirmed that pembrolizumab plus ipilimumab regimen is tolerable and exerts anti-tumor activity in melanoma patients who have progressed after treatment with an anti-PD-1 mAb [90].

Despite these promising clinical data, in about 40–60% of cases resistance to ICI therapy and tumor relapse within 2 years are experienced [91]. This occurs mainly in patients with large tumor burden, high lactate dehydrogenase levels, uveal melanoma, mucosal melanoma, brain metastases and melanoma unresponsive to anti-PD-1 therapies. For this reason, several ongoing clinical trials are investigating novel and hopefully more effective immunotherapeutic combination strategies [92].

## 5. Involvement of Tumor-Associated Macrophages in Melanoma Immune Escape

Among the different sophisticated mechanisms identified in melanoma aimed at establishing an immunosuppressive TME, the release by cancer cells of molecules that can recruit and polarize pro-tumor M2 macrophages seems to play a crucial role.

CSF-1 is an example of molecule with an established role in controlling proliferation, differentiation and survival of macrophages [93] that is involved in melanoma progression and immune escape. Actually, CSF-1 plasma levels in patients with stage III or IV cutaneous melanoma are higher compared to healthy individuals and levels detected in stage IV patients are higher than those in stage IIIB [94]. Accordingly, CSF-1 receptor (CSF1R) signaling in TAMs favor the acquisition of an immunosuppressive M2-like phenotype [95,96]. By quantifying the expression of CSF-1, CSF1R, CD163 (a M2 marker) and CD8 on histological sections of human primary tumors and cutaneous metastases samples, it was demonstrated that tumor areas with high density of tumor-infiltrating CD8+ T-cells were also rich in CSF-1+ tumor cells and CSF1R+/CD163+ TAMs, while in regions where the infiltrated CD8+ T-cell population was limited, scarce M2-TAMs were revealed [94]. These observations suggested the triggering by CD8+ T-cells of a feedback mechanism directed to suppress their overactivation, through the secretion of IFN-γ and TNF-α, which induce CSF-1 production in cancer cells. Thus, activated CTLs drive the recruitment of macrophages to the tumor mass and their M2 polarization, which in turn contribute to limit the anti-tumor immune response.

The previously mentioned marker of M2-TAMs, i.e., CD163, is another important indicator of poor prognosis in melanoma [97,98,99]. CD163 is a macrophage- and bone marrow-derived monocyte-specific transmembrane protein whose expression is induced by tumor promoting cytokines, such as IL-6 and IL-10, and reduced by inflammatory stimuli, including TNF-α and IFN-γ [100,101]. A recent study demonstrated that the specific targeting of a subset of M2-TAMs expressing CD163 induced tumor regression in a murine melanoma model (C57BL/6J mice subcutaneously injected with the anti-PD-1 resistant YUMM1.7 cell line). In fact, the specific depletion of CD163+ TAMs, by using anti-CD163 conjugated lipid nanoparticles, loaded with the anticancer drug doxorubicin (α-CD163-dxr), led to an increased recruitment of effector T-cells. Overall, a significant increase in the tumor-infiltrating leukocytes, from 5 to 30%, was detected, mainly represented by lymphocytes, including CD4+ and CD8+ T-cells and bone marrow-derived monocytes. In contrast, other immune cell subsets (e.g., DCs, B-cells and NK cells) were unaffected. In accordance, the expression of IFN-γ was dramatically elevated in the TME, as well as the expression of other inflammatory cytokines, including TNF-α, IL-1β, and IL-18 [102]. Of note, anti-PD-1 treatment alone did not affect the growth of YUMM1.7 tumors, which are refractory to this ICI. When combined with an anti-PD1 (RMPI-14 clone, 250 µg/mouse), the α-CD163-dxr controlled tumor growth to the same extent observed without anti-PD1 treatment; furthermore, if the αCD163-dxr was replaced with the anti-PD1 therapy, tumors growth quickly resumed, suggesting that depletion of CD163+ M2-TAMs-induced tumor regression independently of the blockade of PD-1/PD-L1 [102].

IL-1α and IL-β are other highly expressed signaling molecules in the TME of primary melanoma tissues [103]. During melanoma progression, the stimulation of their receptor (IL-1R) in M2-TAMs was found to up-regulate the expression of the DNA methylcytosine dioxygenase Ten-Eleven-Translocation-2 (Tet2), a well-known tumor suppressor exerting immunosuppressive functions in hematopoietic malignancies. In particular, Tet2 transcript was found to be increased in TAMs, both in murine in vivo melanoma models (subcutaneous injection of YUMM1.7 and B16-OVA cell lines) and in human peripheral blood and melanoma tissue samples [104]. Deletion of Tet2 in tumor-infiltrating myeloid cells of melanoma-bearing mice (Mye-Tet2 null mice) led to a significant suppression of in vivo tumor growth, compared to wild-type mice. Furthermore, RNA-seq analysis showed that TAMs from Mye-Tet2 null mice overexpressed genes associated with the M1 pro-inflammatory signature, while typical genes of M2 immunosuppressive macrophages, among which Arg-1, were down-modulated, compared to TAMs from wild-type tumor-bearing mice. As expected, when CD4+ T-cells were co-cultured with wild-type TAMs, a marked inhibition of T-cell proliferation was observed, while a significantly reduced suppressive capacity was revealed in T-cells co-cultured with Tet2 null TAMs. Overall, a complete shift of the intratumoral immune compartment towards an anti-tumor environment was established in Mye-Tet2 null mice: the levels of both CD4+ and CD8+ T-cells increased, whereas the percentage of the immunosuppressive Tregs was mildly decreased. Consistently, antibody-based depletion of CD4+ and CD8+ T-cells (by intraperitoneal injection of an anti-CD8 mAb, 53-6.72 clone, and an anti-CD4 mAb, GK1.5 clone, 400 µg each, every 7 days from day 7 after cancer cells injection) rescued the reduced tumor growth phenotype in Mye-Tet2 null mice, thus overcoming the blockade of the immunosuppressive activity of TAMs [104].

Another TAMs-mediated mechanism, through which melanoma can evade the immune system, is based on a deregulated metabolic activity, i.e., on an increased glycolysis, leading to a high release of organic acids, such as lactic acid and pyruvic acid [105], as well as of H^+^ ions, resulting in a typically acid TME. In a preclinical syngeneic animal model, represented by mice injected with the B16F10 melanoma cell line, it was shown that the tumor niche acidification was perceived by macrophage G protein–coupled receptors (GPCR), activating adenylyl cyclase and, therefore, increasing the production of cAMP. In turn, cAMP activated the expression of inducible cAMP early repressor (ICER), known to inhibit the Toll-like receptor (TLR)-dependent NK-κB signaling, which is involved in the pro-inflammatory M1 macrophage polarization [106,107], thus promoting the transition towards the M2 non-inflammatory phenotype. Accordingly, inhibition of cAMP de novo synthesis by the adenylyl cyclase inhibitor MDL-12 abrogated the acidic pH-induced expression of ICER mRNA. Analysis of the RNA isolated from B16F10 melanoma-derived TAMs and bone marrow-derived macrophages (BMDMs) cultured in acidic conditions, showed an overlap of approximately 3% of all the at least two-fold up-regulated genes. Of interest, the transcripts of genes associated with a non-inflammatory macrophage phenotype, such as Arg-1, Clec10a, VEGF-A and Hif-1α, were the most abundant among the up-regulated genes [108]. To establish whether acidosis-induced expression of ICER in TAMs contributed to melanoma immune evasion, mice harboring ICER-deficient macrophages were inoculated with B16F10 melanoma or MC38 colon adenocarcinoma cells. Although no significant differences were observed in the growth pattern of colon adenocarcinoma between ICER-deficient and control mice, animals harboring ICER-deficient macrophages were efficiently able to control and limit B16F10 melanoma growth. In melanoma samples from ICER-deficient mice, an enhanced pro-inflammatory, M1-like polarization (high iNOS and TNF-α expression) was detected, with no variation in the total number of tumor-infiltrating macrophages. Moreover, while no significant changes were observed in the percentage of infiltrating Tregs between B16F10 and MC38 transplanted mice bearing ICER-deficient macrophages, only in the melanoma model an enhanced IFN-γ and TNF-α production by tumor-infiltrating CTLs was observed. In an effort to translate these findings into potentially innovative therapeutic strategies, the de novo synthesis of cAMP was prevented by peritumor injection of MDL-12, in B16F10 and MC38 inoculated C57BL/6J wild-type mice harboring palpable tumors. Noticeably, this in vivo treatment resulted in a significant growth delay of melanoma, but not of colon adenocarcinoma [108].

For what concern amino acid metabolism, the pro-tumor and anti-immunity function of ARG-1, based on arginine depletion, has been assessed in C57BL/6 mice after subcutaneous inoculation of B16 murine melanoma cells (and other syngeneic murine cancer models) and administration of the small-molecule arginase inhibitor CB-1158 (also indicated as INCB001158). Treatment with CB-1158 (100 mg/kg, twice a day) significantly inhibited melanoma growth, compared to the drug vehicle. Conversely, the efficacy of CB-1158 in reducing tumor volume was limited after depletion of either CD8+ or NK cells, thus indicating that certain immune cell populations are required for the full anti-tumor activity of the ARG-1 inhibitor in this cancer model. Consistently, in the same murine melanoma model, CB-1158 treatment resulted in an increased infiltration of the TME by activated CD8+ CD25+ cytotoxic T-cells, compared to vehicle-treated animals [109].

## 6. Involvement of Tumor-Associated Macrophages in Melanoma Resistance to Immune Checkpoint Inhibitors

The established role of M2 macrophages in promoting tumor progression through modification of the TME (by stimulation of angiogenesis, tumor cell motility and immune escape) may play a central role in negatively affecting melanoma response to ICIs. Consequently, approaches aimed at depleting or reprogramming M2-TAMs were proposed to circumvent the failure of therapies based on immune checkpoint blockade and to synergize with ICIs in different murine cancer models [110,111].

**Figure 3:**
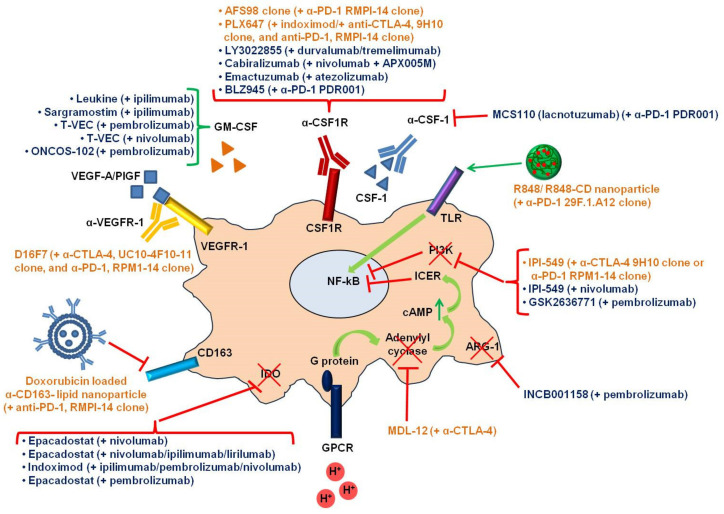
Recent strategies aimed at targeting TAMs in combination with ICIs for melanoma treatment. The schematic drawing illustrates agents, evaluated in preclinical studies (brown) or clinical trials (blue) for melanoma treatment, acting through agonistic (green arrows or bracket) or antagonistic (red blunted arrows or brackets) mechanisms, in combination with anti-PD-1/PDL-1 or anti-CTLA-4 mAbs. GM-CSF agonists, CSF-1 antagonists and CSF1R inhibitors hamper a signaling pathway involved in M2-TAMs recruitment and polarization. IDO and ARG-1 inhibitors counteract depletion of tryptophan and arginine reservoir, respectively, both required for T-cell activity. The adenyl cyclase is a feasible target of anti-TAMs approaches since it inhibits TLR dependent pro-inflammatory NF-kB signaling, by increasing cAMP levels and promoting ICER expression. The same signaling pathway is negatively regulated by PI3K, thus justifying the experimental use of molecules targeting PI3K-γ. Consistently, another TAMs reprogramming pharmacological approach is represented by TLR agonists. Finally, the D16F7 mAb, directed against VEGFR-1, counteracts a signaling pathway involved in M2-TAMs chemotaxis and recruitment to the TME. See text for further details.

To investigate whether M2-TAMs may reduce melanoma responsiveness to ICIs, several studies have been performed in a preclinical setting, by combining TAMs targeting agents and ICIs (Figure 3). An interesting investigation was performed on the SM1 murine melanoma cell line, known to secrete high amounts of CSF-1, and induced to express the highly immunogenic protein ovalbumin (SM1-OVA) [94]. Once inoculated in syngeneic C57BL/6 mice and after the establishment of macrophage-rich tumors, the combined CSF1R and PD-1 blockade (using the rat anti-CSF1R mAb, AFS98 clone, 50 mg/kg, plus the rat anti-PD-1 mAb, RMPI-14 clone, 10 mg/kg, three times a week) was shown to induce a complete regression of all tumors, compared to the single agents. In fact, the sole CSF1R blockade slightly delayed tumor growth, and the PD-1 blockade induced tumor regression in some, but not all animals. Flow cytometric analysis showed a significant reduction of MRC1+ (M2-like) TAMs in mice treated with the anti-CSF1R mAb, and an increase of CD4+ and CD8+ T-cells in the spleens of mice treated with the anti-PD-1 mAb or with both mAbs. These results were confirmed in another melanoma model represented by mice injected with the YUMMER1.7 cells, which derive from YUMM1.7 cells after subsequent rounds of UV-B irradiation to induce de novo mutations [112]. This experimental model better recapitulates the high mutational load of human melanoma and is sensitive to anti-PD-1 agents. Also in this case, a constitutive expression of CSF-1 was revealed and single CSF1R blockade was found to exert a non-significant growth-inhibitory activity, although M2-like MRC1+ TAMs were markedly decreased. The anti-PD-1 treatment alone, instead, inhibited YUMMER1.7 growth in most of the mice, but it did not prolong survival after mAb discontinuation. Of note, the combination of anti-CSF1R and anti-PD-1 mAbs completely eradicated most of the tumors and significantly extended animal survival, indicating strong additive effects for the double targeting regimen [94].

The combination of CSF1R and IDO inhibitors (PLX647 and indoximod, respectively) has been proposed as another approach that was found to elicit tumor regression by stimulating T-cell recruitment. In an IDO overexpressing melanoma murine model (B16-IDO cells transplanted in C57BL/6J mice), this combined treatment [PLX647 incorporated into rodent chow (800 ppm chow) plus indoximod, either dissolved in methylcellulose and administered in drinking water, 2 mg/mL, for a total of 4.5–5.5 mL/day, or administered as implantable subcutaneous pellets, 140 mg/pellet, 14-day-release)] increased mice overall survival and sensitivity to ICIs. In detail, IDO overexpression induced the recruitment of myeloid-derived suppressor cells (MDSCs), able to suppress CD8+ T-cell proliferation in vitro, and conferred resistance to anti-CTLA-4 and/or anti-PD-1 immunotherapy [113,114]. CSF1R blockade with PLX647, instead, depleted suppressive MDSCs and reduced the tumor promoting activity of M2 macrophages, delaying tumor growth only in the B16-IDO melanoma model, but not in control B16. In other words, by targeting MDSCs through CSF1R blockade, it was possible to rescue the immune suppression caused by IDO expression. Furthermore, CSF1R targeting sensitized the tumors to anti-CTLA-4 (100 µg/mouse, 9H10 clone) and anti-PD-1 (250 µg/mouse, RPM1-14 clone) mAbs [114].

In an effort to re-educate the tumor-sustaining M2-TAMs into tumoricidal M1 cells, other small-molecule inhibitors of kinase receptors that mediate signal transduction pathways in TAMs have been tested. An important polarizing effect was observed for the Toll-like receptor 7 and 8 (TLR7/8) agonist R848 (resiquimod), which yielded an M1 enrichment similar to that caused by the standard M1 inducing agents LPS and IFN-γ. In vitro re-polarized murine macrophages by R848 closely resembled M1 controls, and similar results were observed also in human cells [115]. R848 encapsulation within β-cyclodextrin nanoparticles (CDNPs) further increased the TLR7/8 agonist delivery to TAMs and improved re-polarization of macrophages in vivo. Results were obtained using two different therapeutic regimens: repeated dosing (R848, 2.0 mg/kg and CDNP, 16.5 mg/kg, three times weekly) and single dosing (R848, 3.0 mg/kg and CDNP, 24.6 mg/kg) schedules. Importantly, the combination of CDNPs-R848 with an anti-PD-1 mAb (29F.1.A12 clone, 200 µg on day 8–9 following tumor challenge) was synergistic and resulted in a significant tumor reduction in C57BL/6 mice bearing B16F10 melanoma. These results indicated that a nanotherapeutic approach directed against TAMs can sensitize the TME toward combination therapies with ICIs [116].

The activation of phosphoinositide 3-kinase (PI3K) signaling in macrophages promotes immune suppression by inhibiting the pro-inflammatory NF-kB signaling. Accordingly, in several tumor models, PI3K pharmacological inhibition was able to synergize with ICIs inducing tumor regression. B16F10 melanoma cells transduced with a GM-CSF expression vector (B16-GM-CSF), to obtain tumors more infiltrated by suppressive myeloid cells and less sensitive to immune checkpoint blockade, revealed a significant tumor growth inhibition when exposed to IPI-549, a selective inhibitor of the PI3K-γ subunit (15 mg/kg/day) [117]. In particular, PI3K-γ inhibition switched the macrophage activation status from the immunosuppressive M2-like phenotype to the inflammatory M1-like phenotype. In fact, in tumors exposed to IPI-549 the expression of M2 markers (TGF-β, ARG-1, IDO) was reduced, while the expression of M1 markers (IL-12, iNOS) was increased. Treatment with IPI-549 also led to up-regulation of PD-1 and CTLA-4 expression on CD8+ T-cells, suggesting that combining PI3K-γ inhibition with immune checkpoint blockade could provide additional anti-tumor effects. In fact, in the ICIs resistant B16-GM-CSF model, the combination of either an anti-CTLA-4 (9H10 clone, 100 µg/mouse) or an anti-PD-1 (RPM1-14 clone, 250 µg/mouse) (four doses every third day) with IPI-549 significantly delayed tumor growth, compared to ICIs administered as single agents. With double checkpoint blockade alone only 20% of mice bearing B16-GM-CSF tumor got benefit from treatment. Notably, the addition of the PI3K-γ inhibitor to the combination of anti-CTLA-4 plus anti-PD-1 induced a complete remission in 80% of B16-GM-CSF tumor-bearing mice. Moreover, tumor-free survivors were resistant to tumor re-implantation, indicating the establishment of a long-lasting adaptive immunity.

The vascular endothelial growth factor receptor-1 (VEGFR-1) has been recently proposed as a potential target to enhance ICI efficacy [118,119,120]. The membrane form of this receptor is activated by some members of the VEGF family of growth factors (i.e., VEGF-A, VEGF-B and PlGF) and is known to play an important role in melanoma invasiveness, vasculogenic mimicry, neo-angiogenesis as well as in the mobilization of myeloid progenitors from the bone marrow to the tumor site [121,122]. Human activated M2 macrophages have been shown to express higher levels of VEGFR-1, compared to activated M1 cells, and exposure to the anti-VEGFR-1 mAb D16F7 decreased their chemotactic response to the VEGFR-1 selective ligand PlGF [120]. Consistently, in a murine syngeneic model of melanoma (B16F10 cells injected in B6D2F1 mice), VEGFR-1 targeting with the D16F7 mAb (six doses of 10 mg/kg administered on alternate days) inhibited melanoma growth and tumor infiltration by myeloid cells and, in particular, M2 macrophages, in vivo [120,122]. Furthermore, D16F7 mAb was able to reduce immunosuppressive Tregs and PD-1 positive cells, which may also contribute to restrain ICI efficacy [120]. The TME alterations induced by the anti-VEGFR-1 mAb provided the biological rationale for testing of whether the anti-VEGFR-1 mAb might enhance the anti-melanoma activity of ICIs. Interestingly, the combination of D16F7 mAb with an anti-CTLA-4 mAb (UC10-4F10-1 clone), or with an anti-PD-1 (RMP1-14 clone) (four doses of 10 mg/kg every two days) was more effective in suppressing tumor growth compared to each mAb administered as monotherapy [120]. Treatment with ICIs, as single agents or combined with D16F7 mAb, caused an increased infiltration of melanoma nodules by CD8+ T-cells. Furthermore, the anti-VEGFR-1 mAb significantly raised the CD8+/Tregs ratio when administered with the anti-CTLA-4 mAb. Consistently with these findings, treatment with the multi-targeted VEGFR inhibitor axitinib, which besides VEGFR-1 also inhibits VEGFR-2 and VEGFR-3, (seven doses of 25 mg/kg twice a day.) in combination an anti-CTLA-4 (9H10 clone, four doses of 5 mg/kg on alternate days) resulted in increased anti-tumor activity against B16F1 melanoma cells. This effect was in part attributed to improved antigen-presenting function of intratumoral DCs and reduced immunosuppressive activity of tumor-infiltrating myeloid cells [123]. Interestingly, in a mouse intracranial/orthotopic model of glioblastoma, the mAb D16F7 also induced a 65% increase in median survival time respect to that of control animals [124]. This fact is suggestive of the possible efficacy of the antibody also for the treatment of brain metastases, a frequent complication occurring in patients with advanced melanoma.

For what concerns immune checkpoint receptors expressed on TAMs and compensatory resistance mechanisms to ICIs, VISTA was found to be elevated in melanoma patients treated with ipilimumab, and a high proportion of VISTA+ TAMs showed the immunosuppressive M2 phenotype [58]. Moreover, increased expression of intratumoral VISTA+ lymphocytes was observed in biopsies from metastatic melanoma patients who developed resistance to ICIs (nivolumab or pembrolizumab alone or their combination with ipilimumab) [125]. Finally, VISTA was one of the cancer immunity biomarkers found to be overexpressed in 15.8% of 101 patients with different types of malignancies, including melanoma, by using a clinical-grade RNA sequencing assay. Moreover, the co-overexpression of VISTA, TIM-3 (another alternative immune checkpoint) and CD68 (a macrophage marker) significantly correlated with shorter progression-free survival after anti-PD-1/PD-L1-based immunotherapy [126].

Interestingly, immunostaining of melanoma samples from a patient who developed resistance to nivolumab revealed higher expression of IL-34, compared to the primary tumor, which positively correlated with an increased frequency of CD163+ TAMs and poor prognosis [127]. Actually, IL-34 is a cytokine that may promote M2-polarization by competing with M-CSF for the binding to CSF1R [128].

## 7. Clinical Trials Combining Immune Checkpoint Inhibitors and Tumor-Associated Macrophages Targeting Agents

Data obtained from preclinical studies provided a strong rationale for clinical trials testing removal/re-polarization of immunosuppressive macrophages to overcome resistance to ICIs and/or enhance their anti-tumor activity. Several studies combining ICIs with immunomodulatory molecules [13,129], resulting in inhibition of M2-TAMs activity, have been carried out or are currently ongoing in melanoma patients (Table 2 and Figure 3).

Increase of GM-CSF and decrease of M-CSF (CSF-1) levels are examples of practicable and interesting approaches to re-polarize M2-TAMs into M1-TAMs, currently under investigation in combination with ICIs.

In regard to GM-CSF, phase 2 studies are evaluating the safety and efficacy of the recombinant human analogue (sargramostim) combined with ipilimumab, in patients with unresectable stage III or IV metastatic melanoma (NCT01363206; NCT01134614). Interestingly, in the NCT01363206 trial, the median overall survival evaluated from 22 patients was double, compared to that reported for second-line ipilimumab monotherapy (21.1 months vs. 10.1 months) [130]. Similarly, in the NCT01134614 study carried on a total of 245 patients, during a median follow-up of 13.3 months, the reported values of overall survival were 17.5 months (95% CI; 14.9, not reached) and 12.7 months (95% CI; 10.0, not reached) for the combined treatment and ipilimumab, respectively. Moreover, the 1-year survival rate for the ipilimumab plus sargramostim combination was significantly higher than that of ipilimumab alone (68.9% vs. 52.9%); although no difference in progression-free survival was revealed [131], it is undoubting the promising impact of these results. A currently recruiting phase 2/3 clinical trial, with no data available, is testing the side effects of nivolumab and ipilimumab when given together, with or without sargramostim, in patients with stage III–IV unresectable melanoma (NCT02339571). Used as a vaccine adjuvant, sargramostim is also one of the agents used in a still recruiting phase 2 study (NCT04382664) investigating the efficacy and safety of the cancer vaccine UV1, in combination with nivolumab and ipilimumab, as first-line treatment of adult patients with histologically confirmed unresectable or metastatic melanoma. Another recruiting phase 2 clinical trial (NCT02965716), with no reported results, aims at testing the combination of talimogene laherparepvec (T-VEC) plus pembrolizumab in stage III–IV melanoma patients. T-VEC is an oncolytic, recombinant herpes simplex type-1 virus (HSV) encoding human GM-CSF, which selectively infects and replicates in tumor cells, thereby inducing tumor cell lysis. In addition, the encoded GM-CSF may stimulate a cytotoxic T-cell response against tumor cells, resulting in immune-mediated tumor cell death. Thus, T-VEC would convert the TME from an exhausted to a "hot" immune compartment, and might increase melanoma susceptibility to ICIs. Another recent, not yet recruiting, phase 2 study (NCT04330430) will evaluate T-VEC plus nivolumab in the neoadjuvant setting for resectable early metastatic (stage IIIB/C/D-IV M1a) melanoma. Furthermore, an active phase 1 pilot study (NCT03003676) is testing the safety of ONCOS-102, an engineered oncolytic adenovirus expressing GM-CSF, followed by pembrolizumab, in patients with advanced or unresectable melanoma progressing after PD-1 blockade. On June 2019 the sponsor biotechnology company, announced in a press release that clinical responses were observed in 3 out of 9 patients, corresponding to an overall response rate of 33%, in part 1 of this ONCOS-102 trial.

On the other hand, targeting the M-CSF cytokine is expected to result in M2-TAMs depletion and potential increase of ICI activity. This approach has been investigated in a phase 1b/2 study (NCT02807844) assessing the safety, tolerability, pharmacokinetics, pharmacodynamics, and anti-tumor activity of the anti-M-CSF mAb MCS110 (lacnotuzumab), administered in combination with the experimental anti-PD-1 mAb PDR001 (spartalizumab), to adult patients with solid tumors, including melanoma. As reported, the combination was well tolerated overall and anti-tumor activity was observed, in particular in the pancreatic cancer cohort. The most common (≥10%) grade ≥ 3 adverse events were increased aspartate transaminase (12%), asthenia (10%), and hyponatremia (10%), and the most frequent suspected drug-related adverse events were periorbital edema (30%), increased aspartate transaminase (24%) and blood creatine phosphokinase (24%) [132].

The M-CSF receptor (CSF1R) represents another promising target to reduce the immunosuppressive behavior of TAMs and several ongoing or completed clinical trials were designed in order to evaluate the therapeutic potential of combined CSF1R inhibition and ICIs in patients with solid tumors, such as NCT02829723 (CSF1R inhibitor BLZ945 and anti-PD-1 mAb PDR001, recruiting with no data available), NCT02718911 (CSF1R inhibitor LY3022855 and durvalumab or tremelimumab, completed without published results) and NCT02323191 (anti-CSF1R mAb emactuzumab and atezolizumab, completed but no data are available). A currently still recruiting phase 1/1b clinical trial (NCT03502330) is studying the triple combination of nivolumab, cabiralizumab (a humanized mAb directed against CSF1R) and APX005M (a humanized agonistic mAb that binds to CD40 and acts as an immuno-activating agent by triggering the release of IFN-γ), in advanced melanoma, NSCLC and renal cell carcinoma. APX005M is also under evaluation, in combination with nivolumab, in a phase 1/2 study (NCT03123783) aimed at assessing the safety and efficacy of the co-administered treatment in adult subjects with metastatic melanoma (and NSCLC). Interestingly, published results demonstrated that the combination was associated with a good safety profile and a promising anti-tumor activity in melanoma patients with disease progression during previous anti-PD-1 therapy (anti-CTLA-4 therapy was allowed more than 3 months prior to study entry), and the overall toxicity profile was consistent with the profiles of each individual agent [133].

Due to its involvement in T-cell exhaustion, IDO is another interesting target of therapies aimed at avoiding TAMs-mediated immune evasion and resistance to ICIs. A completed phase 1/2 study (NCT02073123) tested the IDO inhibitor indoximod with ICIs (ipilimumab, pembrolizumab and nivolumab) in adult patients with metastatic stage III/IV melanoma. The combination was well tolerated, most common adverse effects being fatigue, nausea, and pruritus. In terms of efficacy, the indoximod plus pembrolizumab regimen demonstrated an overall response rate of 55.7%, favorably comparable with the reported overall response rate for pembrolizumab alone (33%) [134]. A completed phase 1/2 study (NCT02327078) evaluated the safety, tolerability, and efficacy of another IDO inhibitor, i.e., epacadostat, when administered in combination with nivolumab, in various advanced cancer types, including melanoma. As reported, overall response rate was 62% across all patients, while in treatment-naïve patients it was 65%, including both PD-L1-positive and PD-L1-negative patients. The rate of grade ≥3 treatment-related adverse events was 48% with epacadostat higher dose (300 mg, twice a day) and 13% with the lower dose (100 mg, twice a day), allowing to conclude that the combination showed promising anti-tumor activity in patients with advanced melanoma and that the lower dosage was well tolerated [135]. Nevertheless, a completed phase 3 study (NCT02752074) assessing the efficacy and safety of epacadostat plus pembrolizumab, used to treat almost one thousand patients with unresectable or metastatic melanoma, posed some doubts about the usefulness of IDO inhibition as a strategy to enhance the efficacy an anti-PD-1 approach. In fact, the administration of epacadostat plus pembrolizumab twice daily did not significantly improve the progression-free survival and overall survival, if compared with placebo plus pembrolizumab [136]. In another active non-recruiting trial with no shared results (NCT03347123), epacadostat was given in combination with nivolumab and other immunotherapies (ipilimumab or lirilumab), in subjects with advanced or metastatic malignancies, comprising melanoma. Lirilumab is a fully human mAb that binds to the inhibitory receptors KIRDL1/L2/L3 (specifically expressed by NK cells) and avoid their interaction with HLA-C, lowering the threshold for NK cell activation.

Particularly important is the potential role of combined approaches on controlling brain metastases, a very common event that drastically reduces patient’s survival. A phase 2 multicenter clinical trial indicated a promising activity for the combination of ipilimumab and nivolumab also in the central nervous system [137]. An intracranial response rate up to 46% was reported, with higher benefit in patients with asymptomatic untreated brain metastases. However, not all patients obtained substantial benefit from ICI treatment. Importantly, a recent study suggested that IDO enzyme might represent a suitable target in this particular clinical context to enhance the efficacy of ICIs in the brain, being a major product of macrophage/microglia populations infiltrating the TME of melanoma metastases in the central nervous system [138].

As with IDO, ARG-1 is another metabolic enzyme whose inhibition could restore T-cell function, by replenishing arginine storage. A phase 1/2 clinical trial (NCT02903914) is currently testing the efficacy of the ARG-1 inhibitor INCB001158 (or CB-1158), as monotherapy and in combination with pembrolizumab, in patients with advanced/metastatic solid tumors, including melanoma. Results of the ongoing phase 1 study demonstrated that CB-1158 was well tolerated, with no drug-related grade 3 adverse events, and achieved a substantial target inhibition, resulting in increased arginine plasma levels [139].

Given the potential of PI3K inhibition in re-polarizing pro-tumor M2-TAMs into pro-inflammatory M1-TAMs, a phase 1/1b dose-escalation study (NCT02637531) is testing the safety, tolerability, pharmacokinetics and pharmacodynamics of the small-molecule PI3K-γ inhibitor IPI-549, as monotherapy and in combination with nivolumab, for advanced melanoma and other solid tumors. Interestingly, according to first published results, the IPI-549 plus nivolumab combination demonstrated favorable tolerability, early signs of clinical activity, and immune modulation: patients’ blood samples showed evidence of immune activation and reduced immune suppression, in terms of up-regulation of IFN-γ-responsive factors, and dose-dependent proliferation of exhausted PD1+ CD8+ T-cells [140]. A phase 1/2 study (NCT03131908) is also testing the selective PI3K-β inhibitor GSK2636771, in combination with pembrolizumab, in patients with refractory metastatic melanoma characterized by the loss of the tumor suppressor PTEN gene. Safety results are available, suggesting that renal toxicity precludes the higher tested doses; although no objective responses have been observed among the 13 treated patients, two patients experienced a prolonged clinical benefit, and in one case a 27% decrease in tumor burden was obtained [141].

A single completed dose-escalation phase 1 clinical trial (NCT02812875) is testing CA-170, an orally available small molecule designed to target VISTA along with PD-L1 and PD-L2, in patients with advanced solid tumors, comprising also melanoma. The rationale for this study, whose data are unpublished, is that compared to mAbs, small-molecule immune checkpoint inhibitors may offer advantages, in terms of oral bioavailability and lower immunogenicity [142].

## 8. Conclusions

Since their first approval at the beginning of the last decade, therapies based on immune checkpoint blockade have significantly improved the clinical outcome of advanced melanoma. Nevertheless, this tumor can promote the establishment of an immunosuppressive TME in an attempt to evade the host immune responses. Investigation on crucial TME components and immunosuppressive mechanisms that may counteract or limit the efficacy of ICIs is of pivotal importance in order to identify new pharmacological targets aimed at improving the efficacy of immunotherapy for metastatic melanoma.

M2-TAMs represent the major infiltrating leukocyte population within the TME, playing a key role in promoting tumor growth and invasiveness. Preclinical studies have recently suggested that M2 polarized TAMs might be implicated in the failure of immunotherapy-based anticancer treatments. In detail, it has been shown that M2-TAMs express enzymes, receptors, signaling molecules that hamper anti-tumor immunity by impairing T-cell activation. Therefore, targeting M2-TAMs and/or inducing their polarization towards the anti-tumor M1 phenotype, together with the recovery of exhausted T-cells through the co-administration of ICIs, represent a promising approach, currently under investigation in the herein discussed clinical trials.

## Figures and Tables

**Table 1:** Approved ICIs by FDA and EMA.

ICI	Molecular Target	FDA-Approved Indication (Year of Approval) ^a^	EMA-Approved Indication (Year of Approval) ^a^
Ipilimumab	CTLA-4	Melanoma: – adults, metastatic (2011); – BRAF V600 wild-type unresectable/metastatic, in combination with nivolumab (2015); – adjuvant treatment, stage III (2015); – unresectable/metastatic regardless of BRAF mutational status, in combination with nivolumab (2016); – pediatric patients ≥12 years, unresectable/metastatic (2017).	Melanoma: – adults, unresectable or metastatic (2011); – pediatric patients ≥12 years, unresectable/metastatic (2018); – advanced, in combination with nivolumab (2016).
		Renal cell carcinoma: – first-line, intermediate/poor-risk, advanced, in combination with nivolumab (2018).	Renal cell carcinoma: – first-line, intermediate/poor-risk, advanced, in combination with nivolumab (2018).
		Colorectal cancer: – microsatellite instability high (MSI-H) or mismatch repair deficient (dMMR), metastatic, previously treated with a fluoropyrimidine, oxaliplatin and irinotecan, in combination with nivolumab (2018).	
		Hepatocellular carcinoma: – previously treated with sorafenib, in combination with nivolumab (2020).	
		Non-small cell lung cancer (NSCLC) (squamous and non-squamous): – first-line, metastatic, ≥1% PD-L1, without EGFR or ALK mutations, in combination with nivolumab (2020); – first-line, metastatic or recurrent, without EGFR or ALK mutations, in combination with nivolumab and two cycles of platinum-doublet chemotherapy (2020).	NSCLC (squamous and non-squamous): – first-line, metastatic, without EGFR or ALK mutations, in combination with nivolumab and two cycles of platinum-doublet chemotherapy (2020).
		Mesothelioma: – previously untreated unresectable, in combination with nivolumab (2020).	
Nivolumab	PD-1	Melanoma: – unresectable/metastatic and resistant to other agents (2014); – unresectable/metastatic, BRAF V600 wild-type, in combination with ipilimumab (2015); – unresectable/metastatic, regardless of BRAF mutational status, in combination with ipilimumab (2016); – adjuvant, lymph node involvement or metastatic, after completely resection of the tumor (2017).	Melanoma: – unresectable or metastatic, regardless of BRAF mutational status, as single agent (2015) or in combination with ipilimumab (2016); – adjuvant, lymph node involvement or metastatic, after completely resection of the tumor (2018).
		NSCLC (squamous or non-squamous): – metastatic, in progression during or after platinum-based chemotherapy (2015); – first-line, metastatic, ≥1% PD-L1, without EGFR or ALK mutations, in combination with ipilimumab (2020); – first-line, metastatic or recurrent, without EGFR or ALK mutations, in combination with ipilimumab and 2 cycles of platinum-doublet chemotherapy (2020).	NSCLC: – locally advanced or metastatic forms, following prior chemotherapy (2016); – first-line, metastatic or recurrent, without EGFR or ALK mutations, in combination with ipilimumab and 2 cycles of platinum-doublet chemotherapy (2020).
		Small cell lung cancer (SCLC): – metastatic, progressed after platinum-based chemotherapy and at least one other line of therapy (2018).	
		Mesothelioma: – first-line, unresectable, in combination with ipilimumab (2020).	
		Renal cell carcinoma: – advanced/metastatic, previously treated with antiangiogenic therapy (2015); – first-line, advanced, intermediate/poor-risk, in combination with ipilimumab (2018).	Renal cell carcinoma: – advanced, after prior therapy (2016); – first-line, advanced, intermediate/poor-risk, in combination with ipilimumab (2018).
		Classical Hodgkin lymphoma: – relapsed/progressed after autologous hematopoietic stem cell transplantation and brentuximab vedotin and/or ≥3 lines of prior systemic therapy (2016).	Classical Hodgkin lymphoma: – relapsed/progressed after autologous hematopoietic stem cell transplantation and brentuximab vedotin (2016).
		Head and neck squamous cell carcinoma: – recurrent or metastatic with disease progression during or after platinum-based chemotherapy (2016).	Head and neck squamous cell carcinoma: – recurrent or metastatic, with disease progression during or after platinum-based chemotherapy (2017).
		Urothelial carcinoma: – locally advanced or metastatic, in progression during or after platinum-containing chemotherapy or within 12 months from platinum-containing adjuvant or neoadjuvant chemotherapy (2017).	Urothelial carcinoma: – locally advanced, unresectable or metastatic, as second-line treatment, after failure of prior platinum-based chemotherapy (2017).
		Colorectal cancer: – adult and pediatric patients, metastatic with MSI-H or dMMR metastatic, progressed after treatment with a fluoropyrimidine, oxaliplatin, and irinotecan, as a single agent (2017) or in combination with ipilimumab (2018).	
		Hepatocellular carcinoma: – previously treated with sorafenib, as single agent (2017) or in combination with ipilimumab (2020).	
		Esophageal squamous cell carcinoma: – unresectable, advanced, recurrent or metastatic, after prior fluoropyrimidine and platinum-based chemotherapy (2020). combination with ipilimumab (2018).	Esophageal squamous cell carcinoma: – unresectable advanced, recurrent or metastatic, after prior fluoropyrimidine- and platinum-based chemotherapy (2020).
Pembrolizumab	PD-1	Melanoma: – unresectable or metastatic non-responding to previous treatment (2014) and as first-line regardless of BRAF mutational status (2015); – adjuvant, completely resected, with lymph node involvement (2019).	Melanoma: – first-line, unresectable or metastatic (2015); – adjuvant, completely resected, with lymph node involvement (2018).
		NSCLC: – advanced/metastatic, progressed after other treatments and expressing PD-L1 (2015); – first-line, metastatic, high (≥50%) PD-L1 (2016); – first-line, metastatic, non-squamous, in combination with pemetrexed and carboplatin (2017) and without EGFR or ALK mutations (2018), irrespective of PD-L1 expression; – first-line, metastatic, squamous, in combination with carboplatin and either paclitaxel or nab-paclitaxel (2018); – first-line, metastatic or stage III not candidate for surgical resection or definitive chemo-radiotherapy, ≥1% PD-L1 (2019).	NSCLC: – locally advanced or metastatic, after at least one prior chemotherapy regimen, high (≥50%) PD-L1 (2016); – first-line, metastatic, with high PD-L1 expression, without EGFR or ALK mutations (2017); – first-line, metastatic non-squamous, without EGFR or ALK mutations in combination with pemetrexed and a platinum compound (2017); – first-line, metastatic, squamous, in combination with carboplatin and either paclitaxel or nab-paclitaxel (2019).
		SCLC: – metastatic, progressing on or after platinum-based chemotherapy (2019).	
		Head and neck squamous cellcarcinoma: – recurrent or metastatic, progressing on or after platinum-based chemotherapy (2016); – first-line, metastatic or unresectable, recurrent, as monotherapy in tumors expressing ≥1% PD-L1 or in combination with platinum and 5-fluorouracil (2019).	Head and neck squamous cellcarcinoma: – recurrent or metastatic, progressing on or after platinum-based chemotherapy, with high PD-L1 (2018); – metastatic or unresectable, recurrent, as monotherapy in tumors expressing ≥1% PD-L1 or with platinum and 5-fluorouracil (2019).
		Classical Hodgkin lymphoma: – adult and pediatric patients, refractory or relapsed after ≥3 prior lines (2017) or ≥2 prior lines of therapy (2020).	Classical Hodgkin lymphoma: – refractory or relapsed after autologous hematopoietic stem cell transplantation and brentuximab vedotin or who are transplant-ineligible and have failed brentuximab vedotin (2017).
		Urothelial carcinoma: – locally advanced or metastatic, not eligible for cisplatin-containing chemotherapy (as first-line, 2017), ≥10% PD-L1 (2018) or progressing during or following platinum-containing chemotherapy (2017); – high-risk, non-muscle invasive bladder cancer, with carcinoma in situ, with or without papillary tumors, not eligible for cystectomy and unresponsive to Bacillus Calmette-Guérin (BCG) (2020).	Urothelial carcinoma: – locally advanced or metastatic, not eligible for cisplatin-containing chemotherapy (2017), ≥10% PD-L1 (2018) or after platinum-containing chemotherapy (2017).
		Renal cell carcinoma: – first-line, advanced, in combination with axitinib (2019).	Renal cell carcinoma: – first-line, advanced, in combination with axitinib (2019).
		Gastric or gastroesophageal junction cancer: – recurrent, locally advanced or metastatic, ≥1% PD-L1, progressing on or after ≥2 prior lines of therapy with a fluoropyrimidine, platinum-containing and anti-HER2 therapy (2017).	
		Cervical cancer: – recurrent or metastatic, ≥1% PD-L1, progressing on or after chemotherapy (2018).	
		Primary mediastinal large B-cell lymphoma: – adult and pediatric patients, refractory or relapsed after ≥2 lines of therapy (2018).	
		Hepatocellular carcinoma: – previously treated with sorafenib (2018).	
		Merkel cell carcinoma: – adult and pediatric patients, recurrent, locally advanced or metastatic (2018).	
		Esophageal squamous cell carcinoma: – recurrent locally advanced or metastatic, ≥10% PD-L1, progressing after ≥1 line of therapy (2019).	
		Endometrial carcinoma: – advanced, not MSI-H or dMMR, not candidate for curative surgery or radiotherapy, in combination with lenvatinib (2019).	
		Cutaneous squamous cell carcinoma: – recurrent or metastatic, not curable by surgery or radiotherapy (2020).	
		Colorectal cancer: – unresectable or metastatic, progressing after treatment with a fluoropyrimidine, oxaliplatin and irinotecan (2017); – first-line, unresectable or metastatic, MSI-H or dMMR (2020).	
		Solid tumors: – adult and pediatric patients, unresectable or metastatic, MSI-H or dMMR (2017) or high tumor mutational burden (2020) progressing after prior treatment and without satisfactory alternative therapeutic options.	
Cemiplimab	PD-1	Cutaneous squamous cell carcinoma: – metastatic or locally advanced not eligible for curative surgery or radiotherapy (2018).	Cutaneous squamous cell carcinoma: – metastatic or locally advanced not eligible for curative surgery or radiotherapy (2019).
Atezolizumab	PD-L1	Urothelial carcinoma: – locally advanced or metastatic, worsened during or following platinum-containing chemotherapy or within 12 months from platinum-containing adjuvant or neoadjuvant chemotherapy (2016); – locally advanced or metastatic, not eligible for any platinum-containing chemotherapy regardless of PD-L1 expression level (2017) or not eligible for cisplatin-containing chemotherapy, ≥5% PD-L1 (2018).	Urothelial carcinoma: – locally advanced or metastatic, after prior platinum-containing chemotherapy, or cisplatin-ineligible (2017) and ≥10% PD-L1 (2018).
		NSCLC: – metastatic, progressing during or after platinum-containing chemotherapy or, in case of tumors with EGFR or ALK mutation, after prior targeted agents (2016); – first-line, metastatic, non-squamous, without EGFR or ALK mutations, in combination with bevacizumab, paclitaxel and carboplatin (2018); – first-line, metastatic, non-squamous, without EGFR or ALK mutations, in combination with nab-paclitaxel and carboplatin (2019); – first-line, metastatic, high PD-L1 (i.e., 50% of tumor cells or PD-L1 positive tumor-infiltrating immune cells covering ≥ 10% of the tumor area) (2020).	NSCLC: – locally advanced or metastatic, non-squamous, after prior chemotherapy or, in case of tumors with EGFR or ALK mutation, after prior targeted agents (2017); – first-line, metastatic, non-squamous, without EGFR or ALK mutations, in combination with bevacizumab, paclitaxel and carboplatin; if EGFR or ALK mutation are present, the combination with bevacizumab, paclitaxel and carboplatin is administered only after failure of targeted agents (2019); – first-line, metastatic, non-squamous, without EGFR or ALK mutations, in combination with nab-paclitaxel and carboplatin (2019).
		SCLC: – first-line, extensive-stage, in combination with carboplatin and etoposide (2019).	SCLC: – first-line, extensive-stage, in combination with carboplatin and etoposide (2019).
		Triple-negative breast cancer: – unresectable locally advanced or metastatic, ≥1% PD-L1, in combination with nab-placlitaxel (2019).	Triple-negative breast cancer: – unresectable locally advanced or metastatic, ≥1% PD-L1, not receiving prior chemotherapy (2019).
		Hepatocellular carcinoma: – unresectable or metastatic disease, not receiving prior systemic therapy, in combination with bevacizumab (2020).	Hepatocellular carcinoma: – advanced or unresectable carcinoma, not receiving prior systemic therapy, in combination with bevacizumab (2020).
		Melanoma: – BRAF V600 mutation-positive, advanced, in combination with vemurafenib and cobimetinib (2020).	
Durvalumab	PD-L1	Urothelial carcinoma: – locally advanced or metastatic, progressing during or following platinum-containing chemotherapy or within 12 months from platinum-containing adjuvant or neoadjuvant chemotherapy (2017).	
		NSCLC: – unresectable stage III, not progressed after platinum-based chemotherapy and radiotherapy (2018).	NSCLC: – locally advanced, unresectable tumor, ≥1% PD-L1, not progressed after platinum-based chemotherapy and radiotherapy (2018).
		SCLC: – first-line, extensive-stage, in combination with platinum-etoposide (2020).	SCLC: – first-line, extensive-stage, in combination with platinum-etoposide (2020).
Avelumab	PD-L1	Merkel cell carcinoma: – adult and pediatric patients, metastatic, not receiving prior chemotherapy (2017).	Merkel cell carcinoma: – metastatic (2017).
		Urothelial carcinoma: – locally advanced or metastatic disease, progressing during or following platinum-containing chemotherapy or within 12 months from platinum-containing adjuvant or neoadjuvant	
		chemotherapy (2017); – first-line maintenance treatment, locally advanced or metastatic, not progressed following first-line platinum-based chemotherapy (2020).	
		Renal cell carcinoma: – first-line, advanced, in combination with axitinib (2019).	Renal cell carcinoma: – first-line, advanced, in combination with axitinib (2019).

^a^ Data updated to October 2020.

**Table 2:** Clinical trials testing ICIs in combination with TAMs targeting agents for melanoma treatment.

TAMs Targeting Agent	ICI	Tumor	NCT Trial Code ^a^	Phase—Status
GM-CSF agonist				
Sargramostim	Ipilimumab	Unresectable metastatic melanoma	NCT01363206	Phase 2—completed
Sargramostim	Ipilimumab	Stage III–IV unresectable melanoma	NCT01134614	Phase 2—active, non-recruiting
Sargramostim	Nivolumab and ipilimumab	Stage III–IV unresectable melanoma	NCT02339571	Phase 2/3—recruiting
Sargramostim	UV1, nivolumab and ipilimumab	Unresectable or metastatic melanoma	NCT04382664	Phase 2—recruiting
T-VEC	Pembrolizumab	Stage III–IV melanoma	NCT02965716	Phase 2—recruiting
T-VEC	Nivolumab	Resectable early metastatic (stage IIIB/C/D–IV M1a) melanoma (neoadjuvant setting)	NCT04330430	Phase 2—active, non-recruiting
ONCOS-102	Pembrolizumab	Advanced or unresectable melanoma	NCT03003676	Phase 1—active, non-recruiting
M-CSF antagonist				
Lacnotuzumab	Spartalizumab (anti-PD-1 mAb)	Advanced malignancies including melanoma	NCT02807844	Phase 1b/2—completed
CSF1R antagonist				
BLZ945	PDR001	Advanced solid tumors, including melanoma	NCT02829723	Phase 1/2—recruiting
LY3022855	Durvalumab or tremelimumab	Advanced solid tumors, including melanoma	NCT02718911	Phase 1—completed
Emactuzumab	Atezolizumab	Advanced solid tumors, including melanoma	NCT02323191	Phase 1—active, non-recruiting
Cabiralizumab	Nivolumab (and APX005M, CD40 agonistic mAb)	Advanced melanoma, NSCLC and renal cell carcinoma	NCT03502330	Phase 1/1b—recruiting
CD40 Agonist				
APX005M	Nivolumab	Metastatic melanoma and NSCLC	NCT03123783	Phase 1/2—recruiting
IDO inhibitor				
Indoximod	Ipilimumab, pembrolizumab and nivolumab	Stage III/IV melanoma	NCT02073123	Phase 1/2—completed
Epacadostat	Nivolumab	Advanced cancers, including melanoma	NCT02327078	Phase 1/2—completed
Epacadostat	Nivolumab and other immunotherapies (ipilimumab or lirilumab)	Advanced/metastatic malignancies, including melanoma	NCT03347123	Phase 1/2—active, non-recruiting
Epacadostat	Pembrolizumab	Unresectable or metastatic melanoma	NCT02752074	Phase 3, completed
ARG-1 inhibitor				
INCB001158	Pembrolizumab	Advanced/metastatic solid tumors, including melanoma	NCT02903914	Phase 1/2—active, non-recruiting
PI3K inhibitor				
IPI-549	Nivolumab	Advanced solid tumors, including melanoma	NCT02637531	Phase 1/1b—active, non-recruiting
GSK2636771	Pembrolizumab	Refractory metastatic melanoma associated with phosphatase and tensin homolog (PTEN) loss	NCT03131908	Phase 1/2—recruiting

^a^ NCT number or ClinicalTrials.gov identifier; data from https://clinicaltrials.gov, accessed in October 2020.
